# Application of Mathematical and Experimental Approach in Description of Sedimentation of Powder Fillers in Epoxy Resin

**DOI:** 10.3390/ma14247520

**Published:** 2021-12-08

**Authors:** Jakub Smoleń, Piotr Olesik, Jakub Jała, Hanna Myalska-Głowacka, Marcin Godzierz, Mateusz Kozioł

**Affiliations:** 1 Faculty of Materials Engineering, Silesian University of Technology, Krasińskiego 8 Street, 40-019 Katowice, Poland; piotr.olesik@polsl.pl (P.O.); jakub.jala@polsl.pl (J.J.); hanna.myalska@polsl.pl (H.M.-G.); mateusz.koziol@polsl.pl (M.K.); 2 Centre of Polymer and Carbon Materials, Polish Academy of Sciences, M. Curie-Skłodowskiej 34 Street, 41-819 Zabrze, Poland; mgodzierz@cmpw-pan.edu.pl

**Keywords:** epoxy resin, polymer matrix composites, graphite particles, alumina, Stokes’ law

## Abstract

In this paper, sedimentation inhibition attempts were examined using colloidal silica in a mathematical and experimental approach. Experimental results were validated by a two-step verification process. It was demonstrated that application of quantitative metallography and hardness measurements in three different regions of samples allows us to describe the sedimentation process using modified Stokes law. Moreover, proper application of Stokes law allows one to determine the optimal colloidal silica amount, considering characteristics of applied filler (alumina or graphite). The results of mathematical calculations have been confirmed experimentally—the experimental results show good agreement with the calculated data.

## 1. Introduction

Polymer matrix composites (PMC) are widely used in many areas of engineering, e.g., aviation, automotive, wind energy or construction [1,2,3,4,5,6,7,8]. Most engineering PMCs are fiber-reinforced polymer (FRP) [9,10,11], but also polymer concrete [12,13,14] or cast polymers [15,16,17], also known as synthetic cast products. Cast polymers also have a wide range of applications, e.g., cultured marble, cultured onyx or cultured granite used for sinks or pools applications [18,19], or engineered stone for climbing handles [20].

Important in the preparation of slurry for polymers casting is viscosity, reinforcement density and diameter. These factors determine the distribution of reinforcement on a product’s cross-section, mechanical properties and durability of the final product. However, chemical interactions between reinforcement and epoxy might also decrease pot time of resin, depending on the macromolecular structure of resin [21], which might influence the mechanical properties of the composite. To prevent such adverse interactions, there are several ways to obtain a composite with a designed and favorable distribution of reinforcement.

First, chemical modification of resin and/or reinforcement should be mentioned [22]. Application of surfactant or chemical bonders might allow the prevention of reinforcement sedimentation and exclude chemical interactions between reinforcement and resin. However, chemical modifications are expensive and might be applied only for resins with similar chemical structures [23,24,25,26,27]. Due to the large number of resin manufacturers and high sensitivity to the macromolecular structure of resin, it cannot be considered as a common method in the casting of polymer composites.

As a second approach, the application of nano reinforcement should be considered. However, nanoparticles are more expensive than micrometric ones, which strongly impedes their application in the industry [28]. Additionally, most nanoparticles are carcinogenic, due to their low dimensions and high volatility [29,30,31]. Additionally, leading to health and cost issues, nanoparticles have an extremely large specific surface, which might increase other problems related to chemical interactions between reinforcement and resin [32]. On the other hand, its application might strongly increase the mechanical properties of a composite in comparison to conventional reinforcement, even if a lower number of nanoparticles was used [33]. The above description shows that nanocomposites do not undergo the typical sedimentation phenomena, as do composites reinforced with microparticles; therefore, they should be interpreted separately.

One of the best technological methods to prevent reinforcement sedimentation is the application of colloidal silica. Even its low amounts strongly increase the viscosity of the resin, while it has a negligible impact on technological parameters such as pot time or mechanical properties of the final product [34,35]. Considering the economic approach, the application of colloidal silica is well justified.

The mathematical model describing the sedimentation process of inorganic particles added to polymer composites is important and is a practical answer to numerous problems in composites produced by casting techniques. The low viscosity of the resin combined with the long gelling time leads to the intensification of the sedimentation of the fillers. The uneven distribution of particles in the cross-section of the composite material adversely affects the homogeneity of the material and manifests itself in different properties. An additional difficulty in determining the optimal viscosity parameters is the behavior of the resin after adding the hardener, when the viscosity drops rapidly. The temperature has a significant influence on the viscosity. Taking into account a number of phenomena that accompany the hardening process, the development of a mathematical model of sedimentation and the sedimentation threshold beyond which sedimentation is significantly limited is important for many practical aspects of composite production. In the literature [36,37,38,39], numerous attempts can be found to describe the sedimentation of particles in polymer composites, but there is no mathematical model describing the phenomena in the resin. Among the numerous technological problems, sedimentation of fillers is a big problem, especially in the production of composite washbasins, bathtubs, floors, cast machine parts and mold components, etc.

In scientific works, model description of sedimentation has already been undertaken, but a mathematical model for polymer composites reinforced with inorganic particles has never been developed, which is a novelty in this area. The authors of the publication [40] describe the model of the falling velocity of particles in the suspensions. In their experiments, they describe the delaying effect of small particles on the fall of large particles of equal density. Based on the Steinour equation, they calculate the limit velocities and present a simplified model equation for the settlement of particles in a suspension as a function of their free-fall velocity and solids concentration. The paper [41] describes the mathematical model of sedimentation, obtaining the differential equation for the fractional order of particle sedimentation. The authors took into account the fractional origin of the Basset force and, including the fractional Riemann–Liouville integral rewritten as the Grunwald–Letnikov derivative, performed mathematical transformations that allowed for the solution of the fractional differential equation. The proposed model can be used to approximate the sedimentation of fine particles in liquids, the deposition of aerosols in gas flows and the deposition of particles in gas-dispersed systems. Yet, another model approach to the mathematical description of sedimentation was proposed in the article [42], where the authors investigated the sedimentation of identical non-inertial spherical particles in a Stokes fluid within many small particles. It was necessary to take into account changes in viscosity depending on the volume fraction of particles. Saiyad et al. In [43] describes the effect of filler dispersion in a polymer composite for radiation shielding. The authors described radiation shielding in epoxy composites with three types of fillers: graphite, lead and boron nitride nanopowder. The article [44], focused on the preparation and characterization of composites based on biogenic diatomaceous silica and epoxy resins, is also noteworthy. Thanks to the authors’ work, it is possible to obtain information on the influence of the method of obtaining samples and degassing the composite on its mechanical properties. Based on the information contained in the article, it can be found out that the use of silicone molds is more effective in obtaining homogeneous and degassed composites compared to glass molds, which was used in this research.

The main aim of this paper was to propose a repeatable and convenient experimental procedure to determine the optimal amount of silica in epoxy resin, based on Stokes law. Colloidal silica, due to its low density and large specific surface, does not sediment and does not affect the epoxy resin cross-linking process up to 3% by weight [34,45]. The addition of silica to the resin increases its viscosity, but with insufficient weight addition, heavier particles, such as alumina, might still have sediment, which makes the material different at the cross-section. However, in most cases, non-homogeneity is undesirable and must be prevented. The greater the addition of silica, the smaller the sedimentation of the fillers, and above a certain limit, sedimentation does not occur. It is frequent practice to add silica excessively, but this causes numerous technological problems in mixing and also leads to the excessive use of materials.

In further research, a simple two-step verification process was proposed and evaluated on two types of particulate fillers with different densities and mean sizes. The results of the research can be successfully used in many practical aspects of the production of composites, both industrially and scientifically. It should be remembered that the developed model is an approach and does not constitute a universal solution. However, this procedure can be adapted to other cases as well.

## 2. Materials and Methods

In this study, Epidian 505 epoxy resin (Ciech Resins, Sarzyna, Poland) was used, with Z-1 hardener (Ciech Resins, Sarzyna, Poland). Epoxy resin is used for foundry compositions, for laminating, and also for flooring compounds. The resin density at 20 °C is 1.11–1.14 g/cm^3^, its viscosity is about 1500 mPa·s and the gel time is about 40 min (manufacturer data). Two different powders were used as fillers: electro corundum Al_2_O_3_ (supplied by GZMO, Gliwice, Poland) with an average size of D4/3 = 73.2 μm and graphite (supplied by Biomus, Lublin, Poland) with an average size of D4/3 = 44 μm. Hydrophobic colloidal silica Aerosil R202 was supplied by Evonik company (Essen, Germany).

**Figure 1:**
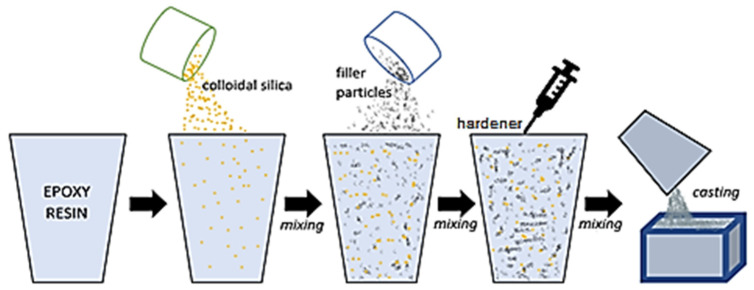
Scheme of making composite samples.

The samples were made by mixing 100 g of resin and a hardener mixture with 20 g of filler powder (temperature 20 °C). Earlier experiments have shown that the 20% filler addition is the most optimal to demonstrate the sedimentation process without adversely affecting the resin crosslinking process. The epoxy mixtures were prepared by using the mass ratio of epoxy-to-hardener (100:10). The composite mixtures containing various amounts of colloidal silica (0.5%, 1%, 2% and 3%) as well as samples without silica were measured using Brookfield DVE LV rotational viscometer (Brookfield Engineering Laboratories, Middleboro, MA, USA) to the determine dynamic viscosity of mixtures. The composites were cast into silicone molds with the base dimensions of 30 mm × 50 mm and a height of 50 mm. The sample production scheme is shown in Figure 1.

**Figure 2:**
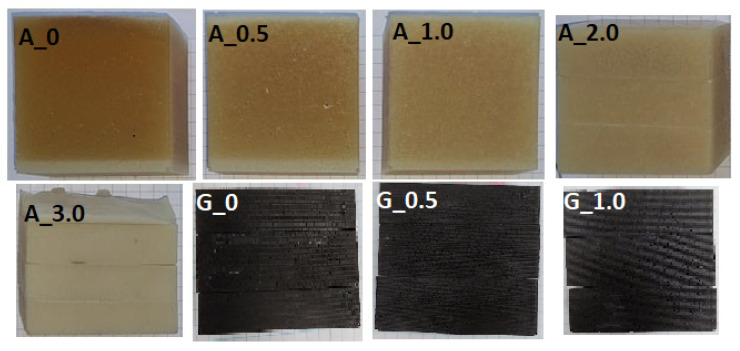
Macrographs of prepared samples.

For the purpose of the research, samples were marked in accordance with Table 1. Macrographs of prepared samples are presented in Figure 2.

Based on the obtained dynamic viscosity measurement results, the viscosity curves were prepared and estimated using exponential regression, according to Equation (1):

(1) $$\eta =ke^{a\%SiO2}$$

where: *k*—flow consistency index [Pa·s^n^], *a*—flow behavior index.

The samples were cut into three sections (top/center/bottom). The surface of alumina-contained composite sample cross-sections were observed using a scanning electron microscope (Hitachi S3000N, Hitachi, Tokyo, Japan) with an Energy Dispersive Spectroscopy (EDS, ThermoNoran, Thermo Electron Corporation, Waltham, MA, USA) in a low vacuum. In the case of samples containing graphite filler, the surface was observed using the light microscopy technique due to the inability to distinguish graphite particles from the epoxy resin using the electron microscope. An acquired cross-sectional image was used in quantitative analysis (QA) for determining the volume fraction of fillers in epoxy resin using Met-Ilo software [46].

The hardness measurements of the samples were performed in three areas of the sample: top/center/bottom, on a HK460 hardness tester (Heckert, East Germany). The results of both QA and hardness measurements were subsequently statically evaluated.

To predict the optimal colloidal silica content, the modified version of Stokes’ law equation was used. In the initial formula (Equation (2)), the particle sedimentation speed is a function of viscosity; hence, it can be presented as a function of colloidal silica content in resin (Equation (3)). However, by assuming desired sedimentation speed in resin, Stokes’ law can be presented as Equation (4). In such a scenario, *V* can be presented as a function of sedimentation distance *h* and resin pot life (gel time) *t_gel_*. In this study, the gel time of raw resin was assumed as 3709 s. The gel time of resin was determined using the Analysis of Temperature Derivative (ATD) method [47]. In the case of sedimentation distance, it was optimized based on quantitative metallography results and linked with the particle mean diameter *d*.

(2) $$V=\frac{1}{18}\frac{\left(\rho_{p}-\rho_{f}\right)}{\eta }gd^{2}$$

(3) $$V=\frac{1}{18}\frac{\left(\rho_{p}-\rho_{f}\right)}{ke^{a\%SiO2}}gd^{2}$$

(4) $$\%SiO2=\frac{ln\;ln\;\frac{\left(\rho_{p}-\rho_{f}\right)gd^{2}}{18hk}}{at}$$

where: *V*—speed of particles sedimentation [m/s]; *ρ_p_*—particles density [kg/m^3^], *ρ_f_*—liquid density [kg/m^3^], *𝜂*—dynamic viscosity [Pa·s], *g*—standard gravity [m/s^2^], *d*—mean diameter of particles [m].

The data collected in the research process and their interpretation allowed for a mathematical description of the filler sedimentation in the composite, which was verified by producing a sample and describing it quantitatively with the SEM/LM technique and hardness testing, showing the correctness of the mathematical model.

## 3. Results

### 3.1. Viscosity Measurements and Stokes’ Law Formula

**Figure 3:**
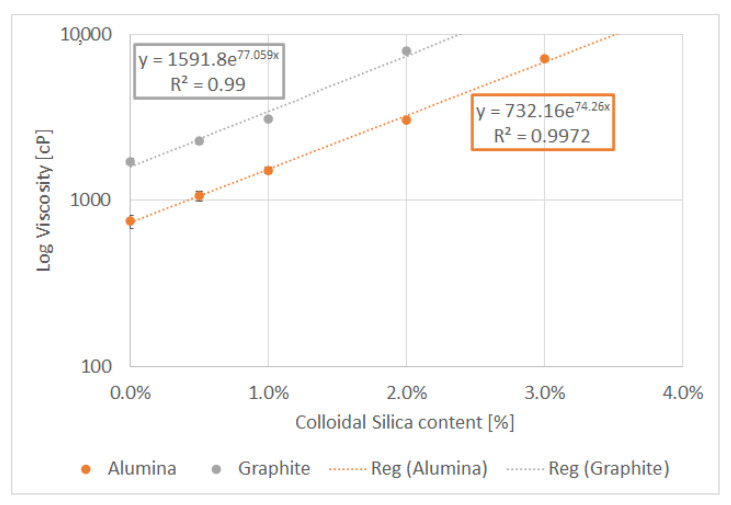
Dynamic viscosity of resins with 20% filler additive (aluminum oxide powder/flake graphite) as a function of colloidal silica additive 0–3%.

The dynamic viscosity results are presented in Figure 3. The graph shows the difference in the initial viscosity of the resins with the addition of aluminum oxide and graphite. The viscosity of the resin with graphite was over two times higher than resin with the same weight amount of alumina. The significant difference in the viscosity of the liquid resin compositions results from the morphology of the powders; the graphite particles are lighter and smaller, which significantly affects the specific surface of the filler. The mass addition of the reinforcement powders is the same, but the volumetric additives are different due to the different densities. Graphite has nearly two times smaller density than Al_2_O_3_, and furthermore, the graphite grains are smaller than the corundum grains, which significantly increases the surface of interaction with the resin, and this manifests itself in a significant difference in viscosity of the two composite systems. The effect of the specific surface of the filler particles on the viscosity of the system is visible and strongly influences the technological process, which is noticeable already at the stage of mixing the resin with the addition of powders. The viscosity of samples with graphite and 3% colloidal silica has not been measured due to its uncastability. Obtained power-law based regressions were used in the modified Stokes’ Law formula.

### 3.2. Sedimentation in Composites with Alumina

**Figure 4:**
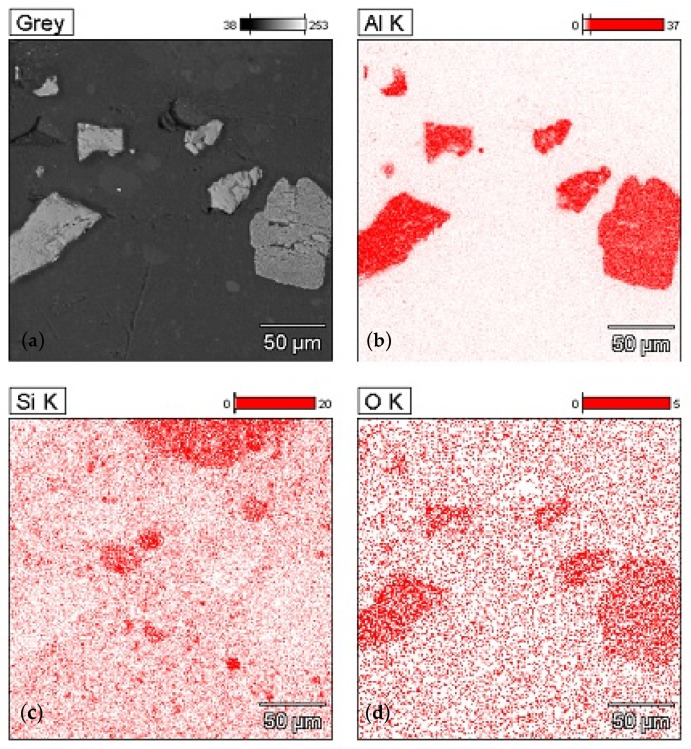
Microstructure and map of the distribution of chemical components in the sample with 20% addition of alumina and the addition of colloidal silica (SEM/EDS technique): (**a**) area of measurement, (**b**) distribution of Al, (**c**) distribution of Si, (**d**) distribution of O.

Figure 4 shows the microstructure and a map of the distribution of chemical elements (aluminum, silicon, oxygen) in a sample with alumina and colloidal silica (EDS, SEM technique). Due to different electron densities and various elemental compositions, acquired SEM micrographs of composites show differences in grayscale contrast. In the image of the microstructure, three types of areas with different contrast can be observed. The brightest areas with irregular shapes are alumina, darker areas with dimensions not exceeding 30 µm are colloidal silica agglomerates, and the dark gray background is the epoxy matrix.

**Figure 5:**
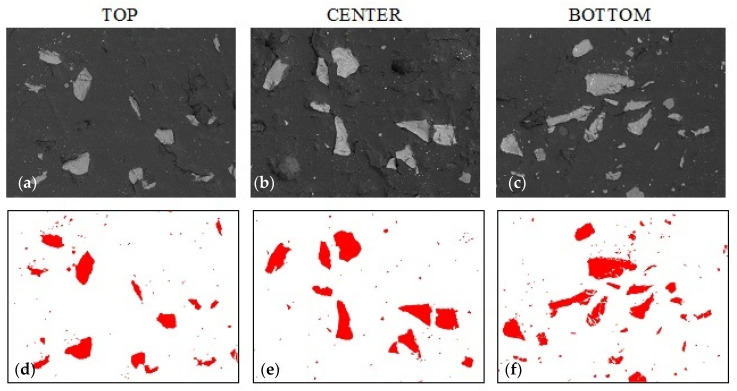
Sample A_0 morphology based on epoxy resin with 20% addition of alumina (0% colloidal silica): (**a**–**c**) sample morphology (SEM technique, magnification 200×), (**d**–**f**) filler distribution (bitmaps after graphic processing).

Figure 5 shows the microstructure of the composite without the addition of colloidal silica with 20% alumina filler content. The top row shows the sample morphology (SEM) in the three regions, while the bottom row shows the distribution of the Al_2_O_3_ filler when the particles are extracted by binarization techniques. The bitmaps created in this way allow the quantitative counting of the percentage of filler particles at the cross-section of the sample. Based on the percentage of the filler, conclusions can be drawn about the sedimentation of particles in the resin volume.

**Figure 6:**
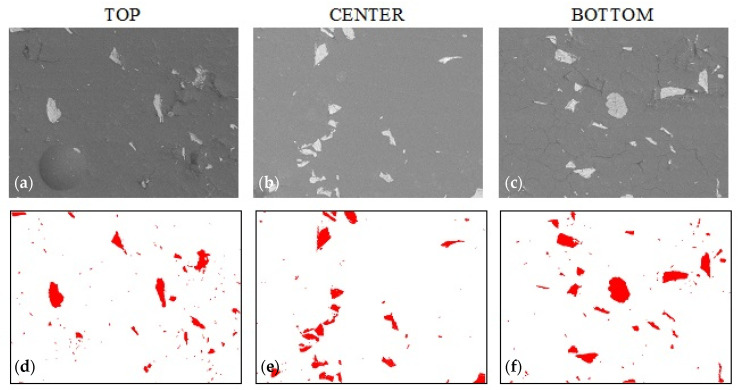
Sample A_1.0 morphology based on epoxy resin with 20% addition of alumina and 1% colloidal silica. (**a**–**c**) sample morphology (SEM technique, magnification 200×), (**d**–**f**) filler distribution (bitmaps after graphic processing).

**Figure 7:**
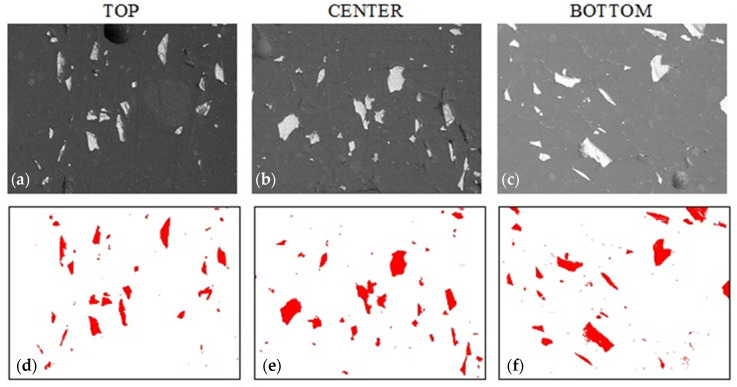
Sample A_2.0 morphology based on epoxy resin with 20% addition of alumina and 2% colloidal silica. (**a**–**c**) sample morphology (SEM technique, magnification 200×), (**d**–**f**) filler distribution (bitmaps after graphic processing).

**Figure 8:**
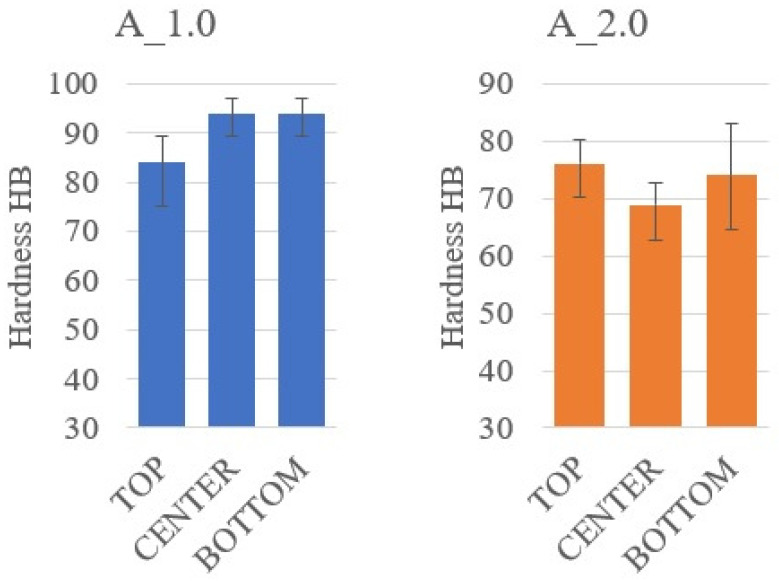
Hardness measurement results for composites with alumina.

Figure 6 and Figure 7 show the samples with 1% and 2% addition of colloidal silica sequentially (sample A_1.0 and sample A_2.0). In Figure 6, sedimentation of the large alumina particles and the stoppage of the smaller particles in the upper part of the sample can be observed. This proves that the sedimentation of particles is partially stopped but is limited only to small particles. It is only with a 2% addition of colloidal silica that the movement of the large alumina particles stops (Figure 7) until the epoxy resin gels, which causes a complete stop of sedimentation. Visual assessment of SEM images does not allow us to determine the differences in the sedimentation process; therefore, using the Met-Ilo software [46], the percentage of filler particles in the cross-section of the sample was determined. Summarized results of QA for samples with 1% and 2% of colloidal silica are presented in Table 2. The statistical evaluation shows that for the sample with 1%, the sedimentation process is visible, while for 2%, the particle’s flow was significantly inhibited. Additionally, in A_1.0, the particles tend to accumulate in the center area. Based on the above results, it can be assumed that the optimal addition of colloidal silica lies in the range of 1–2%. Comparison of these results with Brinell hardness (Figure 8) confirmed the conclusion about inhibited sedimentation in the A_2.0 sample.

### 3.3. Sedimentation in Composites with Graphite

**Figure 9:**
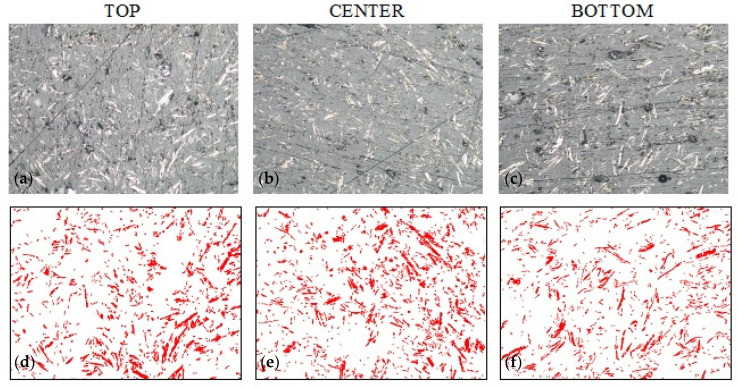
Sample G_0 morphology based on epoxy resin with 20% addition of graphite (0% colloidal silica). (**a**–**c**) sample morphology (SEM technique, magnification 200×), (**d**–**f**) filler distribution (bitmaps after graphic processing).

**Figure 10:**
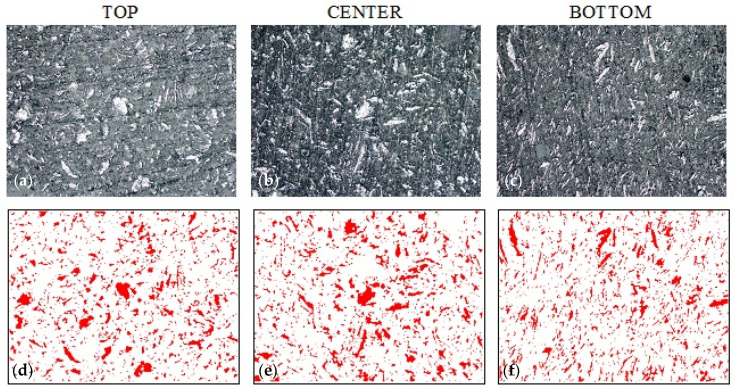
Sample G_1.0 morphology based on epoxy resin with 20% addition of graphite and 1% colloidal silica. (**a**–**c**) sample morphology (SEM technique, magnification 200×), (**d**–**f**) filler distribution (bitmaps after graphic processing).

**Figure 11:**
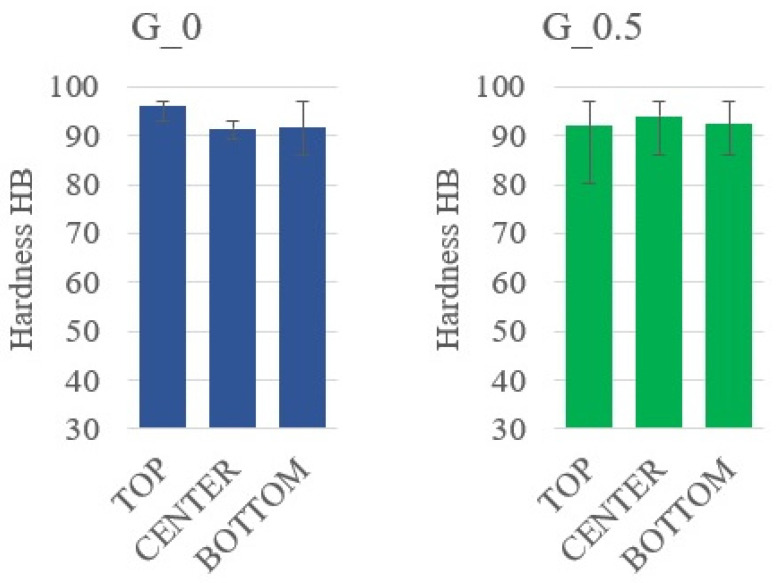
Hardness measurement results for composites with graphite.

A similar methodology for describing the morphology of the samples in the cross-section and determining the percentage of the reinforcing phase in the cross-section of the composite material was conducted in samples G_0 and G_1.0 (Figure 9 and Figure 10). The bright areas are graphite particles, while the dark grey is epoxy matrix. Colloidal silica particles are difficult to see with this observation technique. The statistical evaluation of graphite’s volume content shows minor sedimentation in sample G_0 and a lack of it in samples G_0.5 and G_1.0 (Table 3). Therefore, the range of optimal silica content lies between 0% and 0.5%. However, the results of Brinell hardness (Figure 11) do not confirm sedimentation in G_0. Such results show that in this case, increasing the viscosity with colloidal silica is not necessary from an application point of view. The reason for this may be the unique interaction of graphite particles with resin itself. Hydrophobic graphite flakes are more likely to form a stable suspension with Bisphenol A-based resins than hydrophilic particles such as Al_2_O_3_. Additionally, the higher viscosity of the graphite and resin mixture can additionally hinder the sedimentation.

### 3.4. Stoke’s Formula Modification

Based on obtained data from viscosity measurements, a modification to the Stokes’ law formula was made (Equation (4)). To better visualize the relationship between sedimentation distance h and colloidal silica content %SiO_2_, results for the model were presented in Figure 11. The ratio *h*/*d* describes the sedimentation as a multiplication of powder mean diameter. For example, in the sample A_2.0, “one particle” with 73.2 μm diameter, sediment had a distance of 9582 μm (ratio *h*/*d* = 130), while in A_1.0, sediment had a distance of 20,130 μm (ratio *h*/*d* = 275). In both cases, results from calculation are confirmed at macro graphs (Figure 1), where at A_1.0 there is visible inhomogeneity, while at A_2.0 it cannot be distinguished.

For samples with graphite, modeling results showed that 44 μm-diameter particles’ sedimentation distance was 2640 μm (G_0, *h*/*d* = 60) and 1760 μm (G_0.5, *h*/*d* = 40). Compared to alumina, it is expected that graphite powder, which is less dense and has a smaller grain size, should sediment with a lower distance. With such results, it should be noted that even with different h both in G_0 and A_1.0, the difference in volume fraction between the top and the bottom part was noticed, while in samples G_0.5 and A_2.0, it was not.

**Figure 12:**
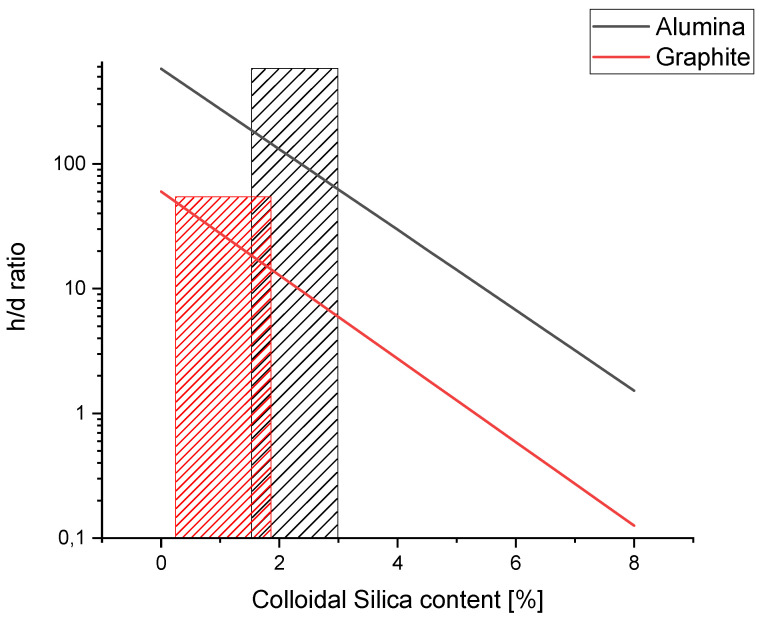
Results of modified Stokes’ law formula with expected area of inhibited sedimentation: red—graphite, black—alumina.

The reason for such a mathematical model mismatch with observation can be explained with established boundary conditions. In these cases, *t_gel_* was assumed to be the same for both compositions (alumina and graphite). However, differences due to the different heat capacities of the used fillers cannot be excluded. Additionally, it was assumed that colloidal silica does not impact a gel time or a curing process. Additionally, the viscosity was assumed to be constant during curing until it reaches the gelation point when it gradually increases. Such factors could lead to a mismatch in sedimentation distance between alumina and graphite. However, based on the empirical results, the *h*/*d* ratio where sedimentation is on an acceptable level was interpolated as *h*/*d* = 200 for alumina and *h*/*d* = 50 for graphite. Such values correspond to colloidal silica content of 1.4% and 0.2% for Al_2_O_3_ and graphite, respectively. According to these values, the area for both inhibited sedimentation and castability of composite composition was established (Figure 12).

### 3.5. Model Verification

**Figure 13:**
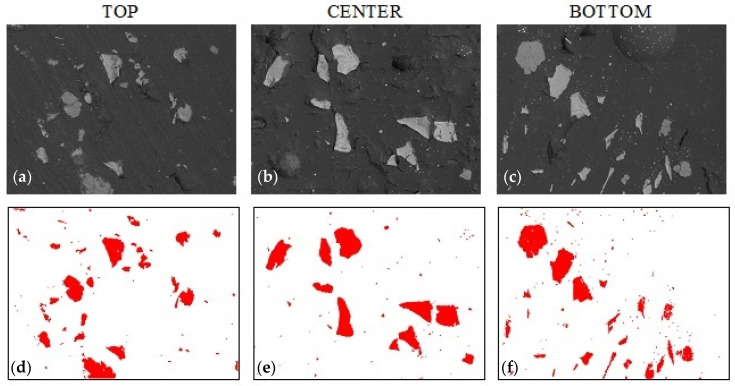
Sample A_1.4 morphology based on epoxy resin with 20% addition of alumina and 1.4% colloidal silica. (**a**–**c**) sample morphology (SEM technique, magnification 200×), (**d**–**f**) filler distribution (bitmaps after graphic processing).

We aimed to verify the developed mathematical model of the inhibited sedimentation process in resins with an assumed gelation time. Due to the hardness results for G_0, only samples for alumina were made. The hypothesis was verified by performing a series of tests in three areas of the sample using the SEM technique and measuring the hardness with the same procedure as before. Results are shown in Figure 13. The quantitative analysis results confirmed the expectations with alumina volume content as follows: 4.74 ± 1.17% (top), 5.29 ± 1.57% (center), and 6.12 ± 1.70% (bottom). Additionally, a hardness test confirmed the structural observation.

## 4. Conclusions

A simple, repeatable, and convenient experimental procedure to determine the optimal amount of colloidal silica in epoxy resin based on Stokes’ law was presented and evaluated in this study. The applied two-step verification process consists of hardness measurements and quantitative metallography, which allows determining the correlation between sedimentation process and colloidal silica content. The following conclusions can be drawn from the research:

1. The sedimentation process has a different nature when different fillers are applied. Various changes in resin viscosity occur as a result of powder density, morphology, size and hydrophobicity. Thus, different amounts of silica content should be applied depending on used powder characteristics. Additionally, the gelation and curing process may affect the sedimentation distance of the filler.
2. The proposed mathematical model based on Stokes’ law allows the optimization of the colloidal silica addition to resin. Such an approach leads to an inhibited sedimentation process. Moreover, that model can be easily modified and applied for various fillers.
3. To obtain more precise values, variables should be considered as functions, i.e., *t_gel_* in relation to filler content, sample size, and ambient temperature.
4. The proposed evaluation methodology, consisting of hardness measurements and quantitative metallography, gives well-correlated results; thus, it can be simplified to only hardness measurements. That improvement allows us to strongly shorten evaluation time and might be applied in cast polymer design and production, due to the simplicity of the measurement procedure and easy interpretation of results.

## Figures and Tables

**Table 1:** List of prepared samples.

Sample Name	Alumina Content	Graphite Content	Colloidal Silica Content
A_0	20%	0%	0%
A_0.5	20%	0%	0.5%
A_1.0	20%	0%	1%
A_2.0	20%	0%	2%
A_3.0	20%	0%	3%
G_0	0%	20%	0%
G_0.5	0%	20%	0.5%
G_1.0	0%	20%	1%
G_2.0	0%	20%	2%

**Table 2:** Volume content of alumina particles in different sample section.

Sample	Top	Center	Bottom
A_1.0	2.62 ± 0.98	5.04 ± 2.83	3.37 ± 0.99
A_2.0	4.35 ± 1.85	4.52 ± 1.42	4.78 ± 1.57

**Table 3:** Volume content of graphite particles in different sample section.

Sample	Top	Center	Bottom
G_0	8.96 ± 1.29	8.80 ± 1.79	11.47 ± 3.12
G_0.5	9.65 ± 1.12	10.20 ± 1.07	10.02 ± 1.33
G_1.0	10.30 ± 1.69	11.46 ± 1.30	10.90 ± 1.32

## Data Availability

Not applicable.
